# Sex Hormone-Binding Globulin and Cardiac Function in Men with Heart Failure: Possible Role of Diabetes

**DOI:** 10.3390/jcm14072132

**Published:** 2025-03-21

**Authors:** Viktor Čulić, Željko Bušić, Riccardo Vio, Tanni Mijić, Ivan Velat

**Affiliations:** 1 Department of Cardiology and Angiology, University Hospital Centre Split, Šoltanska 1, 21000 Split, Croatia; 2 School of Medicine, University of Split, Šoltanska 2A, 21000 Split, Croatia; tanni.mijic1@gmail.com; 3 University Hospital Centre Split-Firule, Spinčićeva 1, 21000 Split, Croatia; zbusic9@gmail.com (Ž.B.); night_shade3@net.hr (I.V.); 4 Department of Cardiothoracic, Vascular Medicine and Intensive Care, Dell’Angelo Hospital, 30174 Mestre-Venice, Italy; riccardo.vio.1@gmail.com

**Keywords:** heart failure, left ventricular diastolic dysfunction, left ventricular ejection fraction, sex hormone-binding globulin, testosterone, type 2 diabetes mellitus

## Abstract

**Background:** The association of sex hormone-binding globulin (SHBG) with heart failure (HF) remains a topic of ongoing debate, particularly in the light of type 2 diabetes mellitus (T2DM). We aimed to assess the association of SHBG with clinical and echocardiographic parameters of HF in men according to the presence of T2DM. **Methods**: Data on baseline characteristics, cardiovascular risk factors and medications, laboratory findings including serum SHBG and total testosterone concentrations, and echocardiographic parameters were prospectively collected for 215 male patients consecutively hospitalized for an acute episode of HF. **Results**: Patients with T2DM were older (*p* = 0.013), had a greater body mass index (*p* = 0.009) and NYHA class (*p* = 0.001), and were more likely to have hypertension (*p* < 0.001) or hyperlipidemia (*p* = 0.032). A moderate correlation among SHBG and total testosterone with the left ventricular ejection fraction (LVEF) was observed only in T2DM patients (r = 0.456) but not among non-T2DM patients (r = 0.194). A multivariate analysis revealed the independent association of increased SHBG levels with lower LVEF values among T2DM patients (ß = −0.542, *p* < 0.0001), whereas in the same group higher total testosterone was an independent predictor of higher LVEF (ß = 0.531, *p* < 0.0001) and lower LVDD (ß = −0.442, *p* = 0.0002) levels. **Conclusions**: In men with HF and T2DM, in contrast to testosterone, SHBG may have an independent adverse impact on the LVEF, which may account for 12.5% of the variance in LVEF levels. The possible subcellular mechanisms of SHBG in men with diabetic myocardial disorder should be additionally explored.

## 1. Introduction

In addition to other clinical symptoms and signs, heart failure (HF) is a clinical syndrome commonly associated with a multiple hormonal imbalance [1]. These imbalances include decreased testosterone [2,3], disturbed thyroid hormone levels [4], insulin resistance, inappropriate antidiuretic hormone secretion [1], and the downregulation of growth hormone [1,5] and insulin-like growth factor-1 [1,6], as well as increased parathyroid hormone and cortisol levels [7].

Sex hormone-binding globulin (SHBG) is a glycoprotein that binds most of the circulating testosterone. Within the context of the hormone imbalance in HF and cardiovascular outcomes, previous studies have reported conflicting results on SHBG, including a lower risk of HF with a low SHBG level [8], a non-linear association with cardiovascular mortality [9], or no association with HF [10]. Recently, increased SHBG has been suggested as a predictor of HF hospitalizations only in dysglycemic men and not in dysglycemic women [11]. Considering cardiac function, only a few studies [12,13] have investigated the association of SHBG with left ventricular (LV) ejection fraction (LVEF), providing limited information on this issue. To the best of our knowledge, there are no data on the association of SHBG with LV diastolic dysfunction (LVDD).

To stress the importance of the independent contribution of type 2 diabetes mellitus (T2DM) to the progression of HF, the term “diabetic myocardial disorder” has been proposed for systolic and/or diastolic myocardial dysfunction in the presence of T2DM [14]. Factors such as a more progressed renal impairment and lower testosterone levels with T2DM may be independent contributors to a worse prognosis of HF in those patients [15]. However, little is known about the possible mechanisms that could mediate the linkages among T2DM, HF, and SHBG. By separating the effect of testosterone, we aimed to assess the independent association of SHBG with clinical and echocardiographic parameters of HF in men according to the presence of T2DM.

## 2. Materials and Methods

### 2.1. Study Patients

Our investigation of the association of serum hormone levels with clinical parameters of HF included men hospitalized due to an acute episode of HF; the findings on thyroid hormones have been reported elsewhere [16]. The present cross-sectional study enrolled 215 male patients, and the inclusion criteria were: (1) clinical presentation typical for HF; (2) LVEF < 50% and/or LVDD established by transthoracic echocardiography; (3) unchanged medical therapy during at least one month. The exclusion criteria were: (1) acute or chronic systemic illness that could affect the hormonal metabolism (i.e., a primary endocrine disorder, liver disease or liver cirrhosis, autoimmune or malignant disease, infection, terminal phase of renal failure, or body mass index (BMI) < 18.5 kg/m^2^); (2) any hormonal treatment or drug intake at the time of the study or in the past (corticosteroids, synthetic thyroid hormones, antithyroid drugs, dopamine, dobutamine); (3) cardiac surgery, acute coronary syndrome, or coronary revascularization within six months before the study; (4) C-reactive protein levels above 15 mg/d. This study complied with the Declaration of Helsinki. The study design and protocol were approved by the Ethics Committee of the University Hospital Center Split (2181-147-10-01/01-M.J.). Each participant provided written informed consent.

### 2.2. Data Collection

The presence of T2DM was defined as previously known diabetes patients on stable therapy for at least 3 months or newly detected T2DM based on an oral glucose tolerance test. For all patients, blood samples for a biochemical analysis and blood count were taken on admission during the initial evaluation in the emergency department. Serum concentrations of SHBG (nmol/L), total testosterone (nmol/L), total triiodothyronine (nmol/L), and plasma N-terminal pro-B type natriuretic peptide (NT-proBNP, pmol/L) were measured using a chemiluminescence immunoassay (Roche Elecsys 2010/Elecsys 1010, Roche Diagnostics GmbH, Mannheim, Germany). Blood samples for the hormones of interest were taken during the first three days of hospitalization between 08:00 and 09:00 to account for circadian variations in circulating hormone levels. For all hormone assays, the intra- and inter-assay variation coefficients were <9% and <13%, respectively.

To measure general adiposity, we calculated the BMI as the body weight in kilograms divided by the squared height in meters. The glomerular filtration rate (GFR, in mL/min/1.73 m^2^) was estimated using the simplified Modification of Diet in Renal Disease formula [17]. Given the minor clinical importance of C-reactive protein levels under 10 mg/L [18], and to account for a low-grade systemic inflammation that accompanies HF [19], the cut-off value for inclusion in the study was set to a maximum of 15 mmol/L, corresponding to a maximum increase of 50% in the C-reactive protein level [20].

### 2.3. Echocardiographic Examination

All subjects underwent a standard transthoracic echocardiographic examination at rest during the first 2 days of hospitalization. The examinations were performed by certified cardiologists using a Vivid 9E device (GE Medical System, Milwaukee WI, USA). All echocardiographic parameters were obtained in accordance with the standard guidelines of the American Society of Echocardiography and the European Association of Cardiovascular Imaging [21]. The LVEF was assessed using the biplane Simpson’s method, whereas the severity of LVDD (3 basic grades) was classified by assessing the early filling (E) and atrial (A) filling peak velocities, E/A ratio, E-wave deceleration time, isovolumic relaxation time, septal and lateral diastolic é and á peak annular tissue velocities, é/á ratio, and LV filling index E/é ratio. The mitral valve inflow for assessment of LVDD was measured on a pulsed-wave Doppler device in the four-chamber view and the sample volume was positioned within one centimeter of the septal and lateral insertion of the mitral valve leaflets [21].

### 2.4. Statistical Analyses

The normally distributed continuous variables are presented as means with standard deviations, and those with a skewed distribution as medians with interquartile ranges. The intergroup differences between those with and without T2DM were tested using the Student’s *t*-test, Mann–Whitney U test, or χ^2^ test where appropriate. The multivariate analysis was deployed by using hierarchical linear regression. To assess the predictive associations of variables of interest for the clinical parameters of HF, the data were entered in 4 blocks (models). In model 1, adjustments were made for age, BMI, and GFR because of their important role in the pathophysiology, presentation, and prognosis of HF, and also because of their independent associations with circulating SHBG levels [22]. Model 2 contained relevant clinical variables and risk factors, model 3 contained serum total testosterone and total triiodothyronine values, and in model 4 SHBG values were added. The change in R^2^ was evaluated for each block and individual contributions of independent variables were calculated by squaring the semi-partial correlation. The predictive values of the independent variables in multiple regression models are expressed using the standardized partial regression coefficient ß and the corresponding *p* value.

All statistical analyses were conducted using IBM SPSS Statistics for Windows, version 26 (IBM Corp., Armonk, NY, USA). A 2-sided α of <0.05 was considered statistically significant.

## 3. Results

The clinical characteristics, laboratory and echocardiographic findings, and medication taken by the study patients according to presence of diabetes are presented in Table 1 and Table 2. The diabetic patients were older, had a greater BMI and NYHA class (Table 1), and were more likely to have hypertension or hyperlipidemia and to take furosemide, calcium antagonists, angiotensin II-receptor blockers, aspirin, or statins (Table 2). All study patients had LVDD with an average grade of 2.5 ± 0.6 (Table 1).

Considering the clinical HF parameters, in the univariable analysis, the SHBG levels inversely correlated with NYHA and NT-proBNP values in the subgroup of patients with T2DM, whereas no significant correlations were observed for echocardiographic parameters of cardiac function or duration of HF (Table 3). In the subgroup of patients without T2DM, no significant correlation was observed between SHBG and any of the clinical or echocardiographic parameters of HF.

Total testosterone positively correlated with the LVEF in both subgroups according to T2DM. Furthermore, in patients with T2DM, testosterone inversely correlated with the LVDD, NT-proBNP, and NYHA class, whereas there was no significant association with the duration of HF. In patients without T2DM, testosterone inversely correlated with NT-proBNP, showed no significant correlation with LVDD or the NYHA class, and showed a borderline significance with the duration of HF (Table 3).

**Figure 1:**
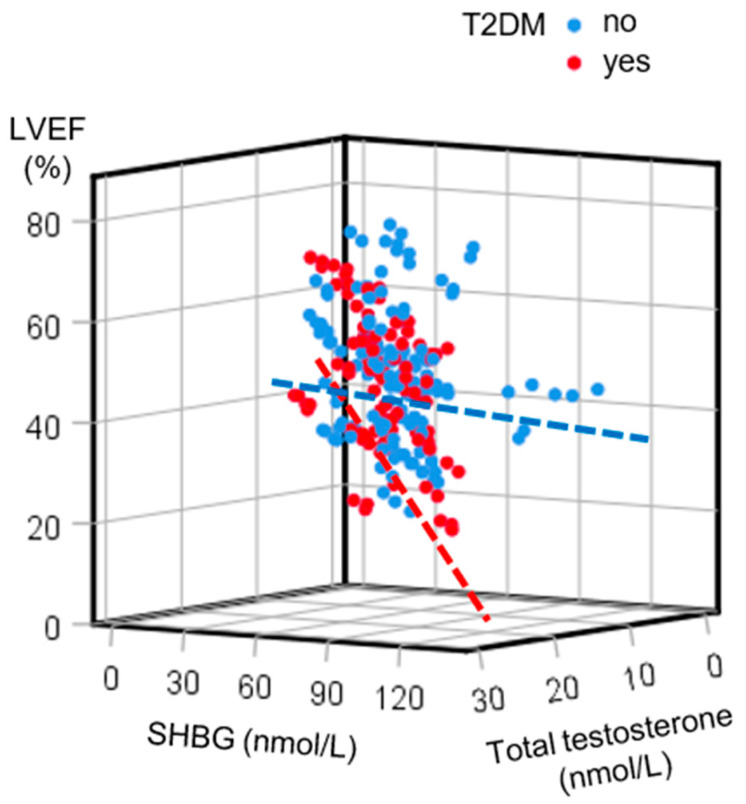
A 3D scatterplot depicting the correlations among sex hormone-binding globulin (SHBG), total testosterone, and left ventricular ejection fraction (LVEF) levels in patients with heart failure according to type 2 diabetes mellitus (T2DM) status. In contrast to a very weak correlation in patients without T2DM (blue dots and dashed line; r = 0.194, linear regression equation LVEF = 42.367 − 0.075 × SHBG + 0.305 × total testosterone), a moderate inverse correlation was observed in patients with T2DM (red dots and dashed line; r = 0.456, linear regression equation LVEF = 45.376 − 0.434 × SHBG + 0.535 × total testosterone).

When plotting a combined relationship among sex hormones and the LVEF, a moderate inverse correlation among SHBG and total testosterone with the LVEF was observed in T2DM patients, whereas a very weak correlation was observed in those without T2DM (Figure 1).

In the multivariable analysis, among the T2DM patients, the adjustments for clinical variables including triiodothyronine and testosterone revealed that SHBG was the most important predictor, contributing 12.5% of the unique variance in LVEF levels in the final model (Table 4). In the same subgroup, SHBG explained 4.6% of the unique variance in the progression of LVDD (Supplemental Table S1). In the final models for the NYHA class (Supplemental Table S2) and duration of HF (Supplemental Table S3), the SHBG levels showed small, non-significant changes of 2.5% and 0.1% from the previous models, respectively.

In patients without T2DM, the SHBG levels showed no significant improvements from the previous models for the echocardiographic and clinical parameters of HF. In the final models, SHBG explained 2.4% of the variance in LVEF values (Supplemental Table S4), 6.9% of the variance in LVDD values (Supplemental Table S5), 1.4% of the variance in NYHA Class values (Supplemental Table S6), and 1.1% of the variance in the duration of HF (Supplemental Table S7).

In patients with T2DM, total testosterone was an independent predictor of higher LVEF and lower LVDD or NYHA values, and showed no predictive association for the duration of HF (Supplemental Table S8). For all of these analyses, there was no significant interaction between testosterone and SHBG (Supplemental Table S8). In patients without T2DM, of the echocardiographic or clinical parameters of HF, testosterone showed a predictive association only with the duration of HF (Supplemental Table S9). In these analyses, a significant interaction between testosterone and SHBG was observed for LVDD, and the interaction for LVEF showed a borderline statistical significance (Supplemental Table S9).

## 4. Discussion

The principal finding of the present study is the adverse effect of increased SHBG with lower LVEF, independent of the serum total testosterone levels, only in male HF patients with coexisting T2DM. In these patients, SHBG may explain 12.5% of the unique variance in LVEF.

Sex hormones have a substantial role in body composition, metabolism, inflammatory processes, and cardiovascular function. In contrast to previous thinking, recent evidence suggests that SHBG is not just a passive protein carrier that simply binds sex hormones, thereby regulating their circulating levels, but that it is a multifunctional protein and mediator involved in a number of physiological and pathophysiological processes, conditions, and diseases [23,24]. SHBG may partly be an inhibitor of sex steroids [12], whereby low levels increase and high levels decrease anabolic activity, favouring catabolic processes [25]. While free testosterone can easily enter the cell via passive diffusion, endocytosis of testosterone bound to SHBG is another means of cellular uptake and partly contributes to the intracellular testosterone activity [26]. The intracellular effects of SHBG are mediated through cell surface signalling, cellular delivery, and the direct activation of specific plasma receptors responsible for sex hormones’ biological actions [27,28]. However, the complex interplay among SHBG, testosterone, and their intracellular mechanisms affecting cardiac function is poorly understood.

The interactions among SHBG, testosterone, and T2DM represent an important facet of this multidimensional problem. A large systematic review with a meta-analysis of observational studies has suggested a link between lower levels of SHBG and a higher risk of T2DM [29]. A somewhat weaker causal interplay of SHBG with insulin resistance and T2DM has been observed in Mendelian randomization studies [29,30]. At the same time, mechanisms linking androgens to the development of T2DM have been well-established; higher total testosterone is associated with a lower T2DM risk in men, whereas in women, conversely, it is associated with an increased T2DM risk [29]. The mediating effect of SHBG in the association between sex and glucose homeostasis, independent of confounders such as age, obesity, and testosterone level, has also been recently reported [31]. Finally, it has been suggested that SHBG may play a more significant role in T2DM than androgens [32].

Data about the association of SHBG with cardiac function in HF are scarce. In men with HF and LVEF values <40%, higher SHBG levels have been associated with an increased 3-year cardiovascular mortality [12]. In men aged 40 to 69 years, independent of total testosterone and other covariates, those with lower SHBG levels had a lower risk for developing HF during a median 9-year follow-up period [8]. In a study using cardiac magnetic resonance to estimate the LV mass, LV mass index, cardiac output, and LVEF, total testosterone showed no significant association with cardiac mass or function after adjustment for clinically relevant variables [13]. In contrast, the associations with SHBG have been weak, explaining less than 1% of the variance in cardiac variables [13]. Compared to that study, our multivariate adjustment included a greater number of clinically relevant variables, and revealed that in the presence of T2DM, a high SHBG level was a predictor of low LVEF (explaining 12.5% of the variance in LVEF) and was associated with a trend toward more progressed LVDD. To the best of our knowledge, this is the first study suggesting that T2DM may play a role in the effects of SHBG on cardiomyocytes of men with HF.

Regardless of the presence of ischemic heart disease or hypertension, the presence of T2DM is associated with a greater likelihood of both LV systolic and LVDD, whereas the echocardiographic abnormalities often include lower LVEF levels, reduced global longitudinal strain, an abnormal E/e’ ratio, LV hypertrophy, and left atrial enlargement [14]. The term diabetic myocardial disorder has been proposed for systolic and/or diastolic myocardial dysfunction in the presence of T2DM. However, T2DM is rarely exclusively responsible for this condition and commonly operates in addition to comorbidities such as arterial hypertension, chronic kidney disease, obesity, or coronary artery disease [14]. While the clinical and echocardiographic features of diabetic myocardial disorder have been defined, the intracellular and metabolic mechanisms that may underlay this condition are less known.

Metabolically, the myocardium is a very active tissue that chiefly uses fatty acids to maintain oxidative phosphorylation and energy production [33]. Changes in cardiac energy metabolism contribute to the pathophysiology, progression, and severity of HF. The coexistence of T2DM independently and substantially influences these processes. In contrast to HF associated with hypertension or ischemia, where myocardial fatty acid oxidation decreases, in HF associated with T2DM fatty acid oxidation increases, making the failing heart even less efficient [34]. Enhanced glucose uptake into the myocardium in the setting of increased fatty acid uptake further contributes to the development of diabetic myocardial disorder. Hyperglycemia and insulin resistance enhance lipid accumulation in the heart, causing lipotoxicity, which is an important mediator of impaired mitochondrial dynamism and cardiomyocyte cytotoxicity [35,36]. These processes promote myocardial collagen deposition, extracellular matrix expansion, and fibrosis, eventually leading to HF [37,38].

A disruption of homeostasis caused by lipotoxicity, mitochondrial dysfunction, and other cardiotoxic processes that accompany T2DM disturb energy production and induce cardiomyocyte death and cardiac fibrosis. Alterations in the metabolic pathways may be associated with transcriptional changes in enzymes responsible for some of these pathways [34]. Whether SHBG, in the presence of T2DM, may influence post-translational epigenetic changes and the expression of genes associated with energy metabolism that may affect the function of cardiomyocytes and LVEF remains to be elucidated. This possibility may be supported by the observation that in men with dysglycemia, increased SHBG levels are associated with a greater risk of HF hospitalizations [11]. Nevertheless, our study suggests a need for future research on the expression of SHBG and its intracellular mechanisms in both healthy myocardial tissue and in HF.

Our study is in agreement with several previous observations that total testosterone may independently affect cardiac function. However, our study also suggests that this effect is confined to patients with T2DM. Chronic inflammation with enhanced production of growth factors, cytokines, and inflammatory mediators that accompany T2DM has been associated with damage caused by disturbed glucose homeostasis [30]. Glucose dysregulation induces metabolic damage and oxidative stress, and through epigenetic mechanisms leads to transcriptional changes [39,40]. In a mouse model, it has been suggested that a reduction in miR-146a, a non-coding RNA, caused by glucose dysregulation in T2DM may be one of the most important mediators of the proinflammatory state responsible for structural and functional cardiac changes, particularly cardiac fibrosis [41]. By opposing such processes, testosterone could be more important for cardioprotection in T2DM patients, with beneficial effects on both the LVEF and LVDD, as suggested by our results. Specifically, the favourable effects of testosterone on the LVEF and LVDD could be mediated through antiproliferative, anticollagen, and antifibrotic properties [2,42,43].

Two earlier meta-analyses of randomized controlled trials of testosterone supplementation within a physiological range in men with chronic HF have shown an improved functional capacity and quality of life expressed through exercise capacity and muscle strength [44,45]. The improvement has not been associated with significant changes in LVEF [44,45]. Nevertheless, it could be explained by effects such as peripheral and coronary vasodilation, an increase in baroreceptor sensitivity, beneficial changes in muscle structure and function, an improved ventilatory response, and an increase in hemoglobin levels and oxygen delivery [2,45]. However, a 2020 updated meta-analysis suggested that significantly improved exercise tolerance is present only with an endpoint of total testosterone of at least 25 nmol/L but not with an endpoint of TT < 25 nmol/L [46]. In terms of major adverse cardiac events, supplementation therapy in men with hypogonadism and a pre-existing or a high risk of cardiovascular disease seems safe [47,48]. Given that male hypogonadism is an important prognostic indicator in HF, this group of patients may be an optimal target population for this therapeutic approach. However, at the moment, there is no consensus about the definition of testosterone deficiency in patients with HF [49,50].

A recent 2024 individual participant data meta-analysis has suggested that lower SHBG levels in men are associated with lower all-cause and cardiovascular mortality, whereas low testosterone is associated with increased all-cause mortality [9]. Therefore, lower SHBG levels coupled with higher testosterone could be a favorable sex hormone profile for cardiovascular and health in general. Our study provides evidence that within this framework, T2DM may play an important independent role by influencing the effects of sex hormones on cardiac function. As described above, men with HF and testosterone deficiency may be a target population for testosterone supplementation. None of the clinical trials investigating testosterone supplementation have investigated the concomitant changes in SHBG levels. Therefore, we can only speculate about the effect of testosterone supplementation on SHBG levels, as well as possible net effects of such hormonal changes on cardiac function. With all that in mind, the most important implication of our study is that in future research, sex hormones in patients with HF should be separately analyzed in those with and those without T2DM.

### Strengths and Limitations

The main strength of our study was the meticulous exclusion criteria designed to avoid potential influence on hormone metabolism. Additionally, we adjusted the associations of interest for a greater number of specific clinical confounders when compared to the previous clinical studies on this topic. To reduce the problem of a close correlation between testosterone and SHBG, and to estimate their separate effects, we standardized the individual values of these hormones and assessed their interactions. The statistical model allowed us to estimate the magnitude of the effects and possible impacts. The main limitation of our study was its observational nature, which prevented us from establishing causal relationships. We did not collect data on glycemic control in patients with T2DM nor on the presence of subclinical coronary artery disease or its extension. Next, we included only male patients with HF, and it is questionable whether our results can be generalized to the female HF population. Finally, we determined serum levels of the investigated hormones, whereas their levels within cardiac tissue remained unknown.

## 5. Conclusions

We found that in men with HF and T2DM, in contrast to testosterone, SHBG may have an independent adverse impact on the LVEF, which may account for 12.5% of the variance in LVEF levels. Our study provides the basis for avenues of future investigation of the potential pathophysiological pathways linking SHBG, testosterone, T2DM, cardiac function, and HF.

## Figures and Tables

**Table 1:** Baseline characteristics and prehospital medication in the study population and in patients according to the presence of T2DM.

	All Patients (*n* = 215)	T2DM Yes (*n* = 91)	T2DM No (*n* = 124)	*p* Value
**Baseline characteristics**				
Age (mean ± SD; years)	74.4 ± 8.0	76.0 ± 7.6	73.3 ± 8.2	0.013
Body mass index (mean ± SD, kg/m^2^)	27.5 ± 5.1	28.5 ± 4.0	26.8 ± 5.6	0.009
LVEF (mean ± SD, %)	46.1 ± 13.7	44.7 ± 13.4	47.2 ± 13.9	0.186
LVDD (mean ± SD, grade)	2.5 ± 0.6	2.6 ± 0.5	2.4 ± 0.6	0.066
NYHA class (mean ± SD, grade)	3.4 ± 0.5	3.6 ± 0.5	3.3 ± 0.5	0.001
HF duration (median, IQR; months)	24 (5–116)	25 (5–157)	16.5 (4–84)	0.076
Arterial hypertension (*n*, %)	123 (57.2%)	70 (76.9%)	53 (42.7%)	<0.001
Hyperlipidemia (*n*, %)	59 (27.4%)	32 (35.2%)	27 (21.8%)	0.032
Previous MI (*n*, %)	44 (20.5%)	22 (24.2%)	22 (17.7%)	0.305
Smoking (*n*, %)	27 (12.6%)	7 (7.7%)	20 (16.1%)	0.062
**Prehospital medication (*n*, %)**				
Loop diuretic	142 (66.0%)	73 (80.2%)	69 (55.6%)	<0.001
Mineralocorticoid receptor antagonist	47 (21.9%)	31 (34.1%)	34 (27.4%)	0.308
Hygroton	6 (2.8%)	3 (3.3%)	3 (2.4%)	0.700
Beta-blocker	114 (53.0%)	53 (58.2%)	61 (49.2%)	0.189
Calcium antagonist	34 (15.8%)	23 (25.3%)	11(8.9%)	0.001
ACEI	88 (40.9%)	36 (39.6%)	52 (41.9%)	0.726
Angiotensin II-receptor blocker	28 (13.0%)	19 (20.9%)	9 (7.3%)	0.003
Aspirin	57 (26.5%)	32 (35.2%)	25 (20.2%)	0.014
Nitrate	13 (6.0%)	6 (6.6%)	7 (5.6%)	0.773
Statin	48 (22.3%)	29 (31.9%)	19 (15.3%)	0.004

T2DM = type 2 diabetes mellitus; SD = standard deviation; IQR = interquartile range; LVEF = left ventricular ejection fraction; LVDD = left ventricular diastolic dysfunction; NYHA = New York Heart Association; HF = heart failure; MI = myocardial infarction ACEI = angiotensin-converting enzyme inhibitor.

**Table 2:** Laboratory findings for the study population and for patients according to the presence of T2DM.

	All Patients (*n* = 215)	T2DM Yes (*n* = 91)	T2DM No (*n* = 124)	*p* Value
Hemoglobin level (mean ± SD; g/L)	129.1 ± 22	122.1 ± 24.9	134.2 ± 18.2	<0.0001
Serum urea (mmol/L)	8.2 (6.1–11.9)	9.5 (7.4–17.4)	7.3 (5.4–9.9)	<0.0001
Serum creatinine (mg/dL)	1.4 (1.1–1.8)	1.5 (1.2–2)	1.3 (1.1–1.5)	<0.0001
GFR (mean ± SD; mL/min/1.73 m^2^)	55.8 ± 19.5	49.7 ± 21.1	60.3 ± 16.9	<0.0001
Urates (mean ± SD; mg/dL)	50.7 ± 152.5	502.7 ± 152.5	492.5 ± 128.6	0.606
High-sensitive troponin (ng/mL)	0.07 (0.02–0.2)	0.1 (0.04–0.4)	0.04 (0.02–0.2)	0.002
NT-proBNP (pmol/L)	479.6 (182–1442)	474.1 (180–1612)	497.4 (194–1271)	0.733
Total testosterone (nmol/L)	10.2 (6–14)	9.1 (6.4–12.8)	11.3 (5.5–14.9)	0.453
Triiodothyronine (nmol/L)	1.3 (1–1.6)	1.2 (0.9–1.5)	1.3 (1–1.6)	0.019
SHBG (nmol/L)	46.6 (32.3–61.9)	43.3 (31–60.6)	47.1 (37.7–65.8)	0.035
Total bilirubin (µmol/L)	18.5 (12.1–27.1)	16.7 (11.4–23.5)	20.8 (12.9–32.1)	0.005
Direct bilirubin (µmol/L)	4.6 (3.1–7.2)	4.2 (2.4–6.2)	5.4 (3.2–8.4)	0.010
Indirect bilirubin (µmol/L)	13.8 (9.1–20)	13.1 (8.5–18.6)	14.5 (9.7–22.6)	0.022
Alkaline phosphatase (U/L)	25 (20–34)	24 (20–32)	27 (20.3–36.8)	0.203
Aspartate aminotransferase (U/L)	24 (18–35)	24 (18–33)	24 (17–37)	0.768
Gamma-glutamyl transferase (U/L)	58 (27–110)	71 (25–114)	50.5 (27.5–107.3)	0.656
Alkaline phosphatase (U/L)	74 (57–100)	78 (68–105)	67 (54–97)	0.008
Total proteins (mean ± SD; g/L)	70.3 ± 8.6	71.2 ± 8.8	69.7 ± 8.4	0.208
Serum albumins (mean ± SD; g/L)	37.5 ± 4.4	37.3 ± 4.6	37.6 ± 4.3	0.649
Serum globulins (mean ± SD; g/L)	32.2 ± 7.6	33.3 ± 8.6	31.3 ± 6.6	0.063
Serum sodium (mean ± SD; mmol/L)	138.1 ± 4.8	137.6 ± 4.9	138.4 ± 4.8	0.273
Serum potassium (mean ± SD; mmol/L)	4.3 ± 0.6	4.4 ± 0.7	4.1 ± 0.6	0.001
Serum chloride (mean ± SD; mmol/L)	99 ± 5.8	99 ± 6	99 ± 5.8	0.997
Serum calcium (mean ± SD; mmol/L)	2.4 ± 0.2	2.4 ± 0.2	2.4 ± 0.2	0.605
Serum magnesium (mean ± SD; mg/dL)	0.8 ± 0.1	0.8 ± 0.1	0.8 ± 0.1	0.133

Values are represented as the median with 25th–75th percentile unless otherwise noted (mean ± standard deviation). T2DM = type 2 diabetes mellitus; SD = standard deviation; GFR = glomerular filtration rate; NT-proBNP = N-terminal pro-B type natriuretic peptide; SHBG = sex hormone-binding globulin.

**Table 3:** Correlation of serum SHBG and total testosterone levels with echocardiographic, laboratory, and clinical parameters of HF in the study population and in patients according to the presence of T2DM.

	All Patients (*n* = 215)	T2DM Yes (*n* = 91)	T2DM No (*n* = 124)
**SHBG**			
LVEF, r (*p*) *	0.03 (0.662)	−0.127 (0.23)	0.087 (0.338)
LVDD, *ρ* (*p*) †	−0.039 (0.567)	−0.029 (0.786)	−0.027 (0.767)
NT-proBNP, r (*p*) *	−0.216 (0.001)	−0.316 (0.002)	−0.146 (0.106)
NYHA, *ρ* (*p*) †	−0.074 (0.281)	−0.329 (0.001)	0.135 (0.146)
Duration of HF, r (*p*) *	−0.089 (0.196)	−0.066 (0.532)	−0.053 (0.557)
**Total testosterone**			
LVEF, r (*p*) *	0.274 (<0.0001)	0.285 (0.006)	0.265 (0.003)
LVDD, *ρ* (*p*) †	−0.225 (0.001)	−0.395 (<0.0001)	−0.135 (0.136)
NT-proBNP, r (*p*) *	−0.418 (<0.0001)	−0.421 (<0.0001)	−0.429 (<0.0001)
NYHA, *ρ* (*p*) †	−0.195 (0.004)	−0.445 (<0.0001)	−0.023 (0.802)
Duration of HF, r (*p*) *	0.119 (0.083)	0.105 (0.321)	0.174 (0.053)
SHBG by total testosterone, r (*p*) *	0.544 (<0.0001)	0.575 (<0.0001)	0.531 (<0.0001)

SHBG = sex hormone-binding globulin; HF = heart failure; T2DM = type 2 diabetes mellitus; LVEF = left ventricular ejection fraction; LVDD = left ventricular diastolic dysfunction; NYHA = New York Heart Association. * Pearson correlation coefficient r and *p* values were obtained from the correlation analysis. † Spearman’s correlation coefficient *ρ* and *p* values were obtained from the Spearman’s rank correlation.

**Table 4:** A hierarchical regression analysis for LVEF in men with HF and T2DM.

Models	R^2^	ΔR^2^	*p* for Change	*p* for Model
1	0.119	0.119	0.012	0.012
2	0.250	0.131	0.009	0.001
3	0.302	0.052	0.055	<0.0001
4	0.426	0.125	<0.0001	<0.0001

LVEF = left ventricular ejection fraction; HF = heart failure; T2DM = type 2 diabetes mellitus; ΔR^2^ = change in R^2^. R^2^, ΔR^2^, and *p* values were obtained from the hierarchical regression analysis. Models: 1—adjusted for age, glomerular filtration rate, and body mass index; 2—adjusted for the variables in model 1 plus history of smoking, blood pressure, previous myocardial infarction, and use of renin–angiotensin–aldosterone system antagonist; 3—adjusted for the variables in model 2 plus triiodothyronine levels and testosterone levels; 4—adjusted for the variables in model 3 plus sex hormone-binding globulin (SHBG) levels. To reduce collinearity between SHBG and testosterone, the values were standardized and the interaction was tested; *p* value for the SHBG × testosterone interaction was 0.75.

## Data Availability

The data sets analyzed in the current study are available from the corresponding author on reasonable request.

## Supplementary Materials

The following supporting information can be downloaded at https://www.mdpi.com/article/10.3390/jcm14072132/s1: Table S1. A hierarchical regression analysis for LVDD in men with HF and T2DM. Table S2. A hierarchical regression analysis for NYHA in men with HF and T2DM. Table S3. A hierarchical regression analysis for the duration of HF in men with T2DM. Table S4. A hierarchical regression analysis for LVEF in men with HF without T2DM. Table S5. A hierarchical regression analysis for LVDD in men with HF but without T2DM. Table S6. A hierarchical regression analysis for NYHA in men with HF without T2DM. Table S7. A hierarchical regression analysis for the duration of HF in men without T2DM. Table S8. Predictive associations of baseline characteristics, risk factors, and circulating SHBG and total testosterone levels for echocardiographic and clinical parameters of HF in men with T2DM. Table S9. Predictive associations of baseline characteristics, risk factors, and circulating SHBG and total testosterone levels for echocardiographic and clinical parameters of HF in men without T2DM.

## Abbreviations

The following abbreviations are used in this manuscript:

HF	Heart failure
SHBG	Sex hormone-binding globulin
T2DM	Type 2 diabetes mellitus
GFR	Glomerular filtration rate
LVEF	Left ventricular ejection fraction
LVDD	Left ventricular diastolic dysfunction
NT-proBNP	N-terminal pro-B type natriuretic peptide
E	Early filling
A	Atrial filling
