# Supplementary material for: Sex Hormone-Binding Globulin and Cardiac Function in Men with Heart Failure: Possible Role of Diabetes
Source: J Clin Med. 2025 Mar 21;14(7):2132. doi: 10.3390/jcm14072132 (PMC11990003; doi:10.3390/jcm14072132)
Supplement: Supplementary file 1 [file jcm-14-02132-s001.zip › jcm-3521670-supplementary.pdf]

Sex Hormone-Binding Globulin and Cardiac Function in Men with Heart Failure: Possible Role of Diabetes

Supplementary material

Supplemental Table S1. Hierarchical regression analysis for LVDD in men with HF and T2DM.

Models	R^2	ΔR^2	p for change	p for model
1	0.065	0.065	0.116	0.116
2	0.166	0.101	0.048	0.03
3	0.239	0.073	0.025	0.006
4	0.285	0.046	0.085	0.003

LVDD = left ventricular diastolic dysfunction; HF = heart failure; T2DM = type 2 diabetes mellitus; ΔR^2 = change in R^2.

R^2, ΔR^2 and p values were obtained from hierarchical regression analysis.

Models: 1 – adjusted for age, glomerular filtration rate and body mass index; 2 – adjusted for the variables in Model 1 plus history of smoking, blood pressure, previous myocardial infarction and use of renin-angiotensin-aldosterone system antagonist; 3 – adjusted for the variables in Model 2 plus triiodothyronine levels and testosterone levels; 4 – adjusted for the variables in Model 3 plus sex hormone-binding globulin (SHBG) levels.

To reduce collinearity between SHBG and testosterone the values were standardized and the interaction was tested; p value for the SHBG*testosterone interaction was 0.297.

Supplemental Table S2. Hierarchical regression analysis for NYHA in men with HF and T2DM.

Models	R^2	ΔR^2	p for change	p for model
1	0.144	0.144	0.003	0.003
2	0.303	0.159	0.002	< 0.0001
3	0.415	0.112	0.001	< 0.0001
4	0.441	0.025	0.173	< 0.0001

NYHA = New York Heart Association; HF = heart failure; T2DM = type 2 diabetes mellitus; ΔR^2 = change in R^2.

R^2, ΔR^2 and p values were obtained from hierarchical regression analysis.

Models: 1 – adjusted for age, glomerular filtration rate and body mass index; 2 – adjusted for the variables in Model 1 plus history of smoking, blood pressure, previous myocardial infarction and use of renin-angiotensin-aldosterone system antagonist; 3 – adjusted for the variables in Model 2 plus triiodothyronine levels and testosterone levels; 4 – adjusted for the variables in Model 3 plus sex hormone-binding globulin (SHBG) levels.

To reduce collinearity between SHBG and testosterone the values were standardized and the interaction was tested; p value for the SHBG*testosterone interaction was 0.101.

Supplemental Table S3. Hierarchical regression analysis for duration of HF in men with T2DM.

Models	R^2	ΔR^2	p for change	p for model
1	0.166	0.166	0.001	0.001
2	0.199	0.033	0.491	0.008
3	0.266	0.067	0.029	0.002
4	0.267	0.001	0.976	0.007

HF = heart failure; T2DM = type 2 diabetes mellitus; ΔR^2 = change in R^2.

R^2, ΔR^2 and p values were obtained from hierarchical regression analysis.

Models: 1 – adjusted for age, glomerular filtration rate and body mass index; 2 – adjusted for the variables in Model 1 plus history of smoking, blood pressure, previous myocardial infarction and use of renin-angiotensin-aldosterone system antagonist; 3 – adjusted for the variables in Model 2 plus triiodothyronine levels and testosterone levels; 4 – adjusted for the variables in Model 3 plus sex hormone-binding globulin (SHBG) levels.

To reduce collinearity between SHBG and testosterone the values were standardized and the interaction was tested; p value for the SHBG*testosterone interaction was 0.874.

Supplemental Table S4. Hierarchical regression analysis for LVEF in men with HF without T2DM.

Models	R^2	ΔR^2	p for change	p for model
1	0.082	0.082	0.016	0.016
2	0.213	0.130	0.001	< 0.0001
3	0.284	0.071	0.005	< 0.0001
4	0.308	0.024	0. 491	< 0.0001

LVEF = left ventricular ejection fraction; HF = heart failure; T2DM = type 2 diabetes mellitus; ΔR^2 = change in R^2.

R^2, ΔR^2 and p values were obtained from hierarchical regression analysis.

Models: 1 – adjusted for age, glomerular filtration rate and body mass index; 2 – adjusted for the variables in Model 1 plus history of smoking, blood pressure, previous myocardial infarction and use of renin-angiotensin-aldosterone system antagonist; 3 – adjusted for the variables in Model 2 plus triiodothyronine levels and testosterone levels; 4 – adjusted for the variables in Model 3 plus sex hormone-binding globulin (SHBG) levels.

To reduce collinearity between SHBG and testosterone the values were standardized and the interaction was tested; p value for the SHBG*testosterone interaction was < 0.0001.

Supplemental Table S5. Hierarchical regression analysis for LVDD in men with HF without T2DM.

Models	R^2	ΔR^2	p for change	p for model
1	0.073	0.073	0.029	0.029
2	0.101	0.028	0.472	0.086
3	0.138	0.037	0.09	0.044
4	0.208	0.069	0.009	0.005

LVDD = left ventricular diastolic dysfunction; HF = heart failure; T2DM = type 2 diabetes mellitus; ΔR^2 = change in R^2.

R^2, ΔR^2 and p values were obtained from hierarchical regression analysis.

Models: 1 – adjusted for age, glomerular filtration rate and body mass index; 2 – adjusted for the variables in Model 1 plus history of smoking, blood pressure, previous myocardial infarction and use of renin-angiotensin-aldosterone system antagonist; 3 – adjusted for the variables in Model 2 plus triiodothyronine levels and testosterone levels; 4 – adjusted for the variables in Model 3 plus sex hormone-binding globulin (SHBG) levels.

To reduce collinearity between SHBG and testosterone the values were standardized and the interaction was tested; p value for the SHBG*testosterone interaction was 0.002.

Supplemental Table S6. Hierarchical regression analysis for NYHA in men with HF without T2DM.

Models	R^2	ΔR^2	p for change	p for model
1	0.11	0.11	0.003	0.003
2	0.171	0.061	0.082	0.003
3	0.179	0.008	0.561	0.006
4	0.193	0.014	0.386	0.01

NYHA = New York Heart Association; HF = heart failure; T2DM = type 2 diabetes mellitus; ΔR^2 = change in R^2.

R^2, ΔR^2 and p values were obtained from hierarchical regression analysis.

Models: 1 – adjusted for age, glomerular filtration rate and body mass index; 2 – adjusted for the variables in Model 1 plus history of smoking, blood pressure, previous myocardial infarction and use of renin-angiotensin-aldosterone system antagonist; 3 – adjusted for the variables in Model 2 plus triiodothyronine levels and testosterone levels; 4 – adjusted for the variables in Model 3 plus sex hormone-binding globulin (SHBG) levels.

To reduce collinearity between SHBG and testosterone the values were standardized and the interaction was tested; p value for the SHBG*testosterone interaction was 0.505.

Supplemental Table S7. Hierarchical regression analysis for duration of HF in men without T2DM.

Models	R^2	ΔR^2	p for change	p for model
1	0.059	0.059	0.063	0.063
2	0.185	0.126	0.002	0.001
3	0.226	0.041	0.054	< 0.0001
4	0.237	0.011	0.976	0.001

HF = heart failure; T2DM = type 2 diabetes mellitus; ΔR^2 = change in R^2.

R^2, ΔR^2 and p values were obtained from hierarchical regression analysis.

Models: 1 – adjusted for age, glomerular filtration rate and body mass index; 2 – adjusted for the variables in Model 1 plus history of smoking, blood pressure, previous myocardial infarction and use of renin-angiotensin-aldosterone system antagonist; 3 – adjusted for the variables in Model 2 plus triiodothyronine levels and testosterone levels; 4 – adjusted for the variables in Model 3 plus sex hormone-binding globulin (SHBG) levels.

To reduce collinearity between SHBG and testosterone the values were standardized and the interaction was tested; p value for the SHBG*testosterone interaction was 0.54.

Supplemental Table S8. Predictive associations of baseline characteristics, risk factors and circulating SHBG and total testosterone levels for echocardiographic and clinical parameters of HF in men with T2DM.

	**LVEF**		**LVDD**		**NYHA**		**HF duration**	
**Variable**	**ß**	**p**	**ß**	**p**				
Age (years)	0.190	0.097	– 0.109	0.39	0.049	0.665	0.229	0.078
Body mass index (kg/m^2)	0.075	0.537	0.046	0.732	0.138	0.25	0.511	< 0.0001
GFR (mL/min/1.73 m^2)	0.049	0.724	0.063	0.682	– 0.431	0.002	0.011	0.943
Smoking	0.206	0.042	– 0.115	0.303	– 0.334	0.001	– 0.047	0.68
Arterial hypertension	– 0.318	0.014	0.003	0.984	0.132	0.293	– 0.228	0.114
Previous MI	0.125	0.199	0.135	0.214	– 0.075	0.432	0158	0.152
RAAS antagonist	– 0.215	0.025	0.319	0.003	0.232	0.014	– 0.042	0.691
Total triiodothyronine (nmol/L)	0.020	0.873	– 0.002	0.986	0.155	0.203	– 0.337	0.017
Total testosterone (nmol/L)	0.531	< 0.0001	– 0.442	0.002	– 0.433	0.001	0.199	0.163
SHBG (nmol/L)	– 0.542	< 0.0001	0.287	0.053	0.125	0.335	– 0.023	0.875
SHBG*testosterone interaction	0.031	0.75	0.114	0.297	– 0.159	0.101	0.018	0.874

SHBG = sex hormone-binding globulin; HF = heart failure; T2DM = type 2 diabetes mellitus; LVEF = left ventricular ejection fraction; LVDD = left ventricular diastolic dysfunction; NYHA = New York Heart Association; GFR = glomerular filtration rate; MI = myocardial infarction; RAAS = renin-angiotensin-aldosterone system.

ß and p values were obtained from the multiple regression analysis, ß = standardized regression coefficient. To reduce collinearity between SHBG and testosterone the values were standardized.

Supplemental Table S9. Predictive associations of baseline characteristics, risk factors and circulating SHBG and total testosterone levels for echocardiographic and clinical parameters of HF in men without T2DM.

	LVEF		LVDD		NYHA		HF duration	
**Variable**	**ß**	**p**	**ß**	**p**				
Age (years)	0.153	0.092	0.011	0.913	0.029	0.763	0.228	0.018
Body mass index (kg/m^2)	– 0.13	0.165	0.3	0.003	0.095	0.346	0.103	0.295
GFR (mL/min/1.73 m^2)	0.258	0.007	– 0.174	0.086	– 0.379	< 0.0001	0.155	0.119
Smoking	– 0.068	0.429	0.141	0.128	– 0.068	0.467	– 0.036	0.69
Arterial hypertension	0129	0.177	– 0.078	0.442	– 0.192	0.064	0.13	0.196
Previous MI	– 0.108	0.193	– 0.107	0.226	0	1	– 0.094	0.28
RAAS antagonist	0.252	0.007	0.077	0.431	– 0.077	0.434	0.279	0.004
Total triiodothyronine (nmol/L)	0.191	0.037	– 0.198	0.043	– 0.029	0.77	– 0.016	0.868
Total testosterone (nmol/L)	0.091	0.416	0.101	0.401	– 0.01	0.934	0.307	0.01
SHBG (nmol/L)	– 0.008	0.94	0.028	0.812	0.125	0. 293	– 0.111	0.336
SHBG*testosterone interaction	0.19	0.055	– 0.324	0.002	0.071	0.505	– 0.063	0.54

SHBG = sex hormone-binding globulin; HF = heart failure; T2DM = type 2 diabetes mellitus; LVEF = left ventricular ejection fraction; LVDD = left ventricular diastolic dysfunction; NYHA = New York Heart Association; GFR = glomerular filtration rate; MI = myocardial infarction; RAAS = renin-angiotensin-aldosterone system.

ß and p values were obtained from the multiple regression analysis, ß = standardized regression coefficient. To reduce collinearity between SHBG and testosterone the values were standardized.
